# Linking Crohn's Disease and Ankylosing Spondylitis: It's All about Genes!

**DOI:** 10.1371/journal.pgen.1001223

**Published:** 2010-12-02

**Authors:** Dirk Elewaut

**Affiliations:** Laboratory for Molecular Immunology and Inflammation, Department of Rheumatology, Ghent University Hospital, Ghent, Belgium; Georgia Institute of Technology, United States of America

Chronic inflammatory arthritis, a hallmark of several inflammatory rheumatic diseases, and inflammatory bowel disease are both life-long conditions, with substantial morbidity and even mortality. These diseases are highly prevalent—for example, chronic arthritis has a frequency of approximately 2%–3% within a given population. Interestingly, the co-existence of gut and joint inflammation was found to be prominent in spondyloarthritis (SpA), a family of interrelated rheumatologic diseases. SpA has a number of typical clinical and genetic characteristics, including peripheral arthritis (particularly of lower limb joints) as well as inflammation of the axial skeleton (e.g., spine). Moreover, different forms of SpA may also affect other organs, such as the skin (psoriasis) or the eye (anterior uveitis), demonstrating the systemic nature of these diseases. Various subtypes of SpA have been described based upon clinical features, but any two may share important characteristics. The prototypical disorder of the SpA family is ankylosing spondylitis (AS), which is characterized by prominent inflammation of the axial skeleton (spine, sacroiliac joints), although other joints may also be affected. Other SpA diseases include infection-triggered reactive arthritis, some forms of juvenile idiopathic arthritis, arthritis in association with inflammatory bowel diseases (IBD), and some forms of psoriatic arthritis [1], [2].

The striking relationship between IBD and AS has been recognized for many years: up to 10% of IBD patients develop AS, and, vice versa, IBD commonly develops in patients primarily diagnosed with AS [3]. As both have an important underlying genetic heritability, it has been suggested that the two diseases could have an overlapping set of predisposing genes. Strong evidence for this idea has been derived from the Icelandic genealogy database: it was shown that AS and IBD have a strong elevated cross-risk ratio in first- and second-degree relatives. However, the precise nature of the predisposing genes remained unknown for some time [4].

In this issue of *PLoS Genetics*, Danoy et al. [5] report on the results of genome-wide association studies looking at a set of recently identified Crohn's disease (CD) susceptibility genes in a large cohort of AS patients [6]. This is the first large-scale study to address the issue of a possible genetic link between CD and AS by using a step-wise approach that includes both an initial exploratory and a confirmatory cohort. New loci associations were identified, including one within the intergenic region at chr1q32, found near the gene *KIF21B*, which encodes a protein of the kinesin motor family involved in transport along axonal and dendritic microtubules. However, a clear-cut association with *KIR21B* itself is not apparent from this study, so undoubtedly more work is needed in this area.

One particularly interesting aspect of the paper is the elucidation of a strong association with genes implicated in the Th17 pathway, a lymphocyte subset that has gathered much attention lately because of its prominent role in a variety of immune-mediated inflammatory disorders, including psoriasis and CD. While the association of AS with the receptor for IL-23, which is implicated in the expansion and survival of Th17 cells, has been previously reported [7], [8], Danoy and co-workers provide two additional links to the Th17 pathway. Firstly, they report a clear association with *STAT-3*, which is, amongst other things, implicated in IL-23R signal transduction. In addition, an association with the p40 subunit shared between IL-12 and IL-23 was revealed. It is intriguing that so many genes predispose to AS. The functional significance of these associations is, however, presently unclear. For example, some of the IL-23R single nucleotide polymorphisms associated with AS may confer either protection or susceptibility to the disease [7]. Nevertheless, more than 30 years after the discovery of HLA-B27 as a strong hereditability factor for AS [9], further evidence points to an important genetic susceptibility for adaptive immunity shared with CD [10].

Danoy et al. also found significant but weaker associations with the LRR2/MUC19 locus. This locus contains genes involved in the process of autophagy and epithelial integrity, respectively.

However, one important limitation of the new study is the potential bias caused by subclinical bowel inflammation. Approximately two-thirds of patients suffering from SpA, including AS, have microscopic signs of gut inflammation without any accompanying clinical gastrointestinal symptoms [11]–[13]. In fact, mucosal alterations are one of the first signs of ongoing inflammation in SpA. Histologically, the gut inflammation can be divided into acute (mimicking a short-term and self-limiting bacterial enterocolitis) and chronic types (with altered intestinal architecture, blunted and fused villi, and influx by mononuclear cells), common in enterogenic-triggered reactive arthritis and AS patients, respectively [12]. Furthermore, 6%–13% of these patients eventually develop IBD, particularly CD. This progression to overt CD is a very peculiar feature of the chronic type of inflammation where up to 20% of those that have chronic gut inflammation develop CD [3], [14]. Thus, even though the results of the present study were not altered significantly by excluding the cases of clinical IBD, it is conceivable that the genetic overlap between IBD and AS may be mirrored by the presence of subclinical gut inflammation. This is particularly the case for the chronic type of inflammation. Previously, a similar genetic link to the chronic subtype of inflammation was found for CARD15 [15], single nucleotide polymorphisms of which are also strongly linked to CD [16]–[18]. However, the frequency of CARD15 variants was not elevated in SpA patients with an acute type of inflammation or in patients lacking signs of mucosal inflammation [15]. Thus, it is clear that more studies are needed linking the presence of subclinical gut inflammation in AS to the association of genes. Other items on the research agenda should include the functional significance of the identified gene polymorphisms in shaping the immune response and the potential interaction between the single nucleotide polymorphism of the various genes identified and their impact on clinical manifestation of disease. It is clear that exciting times lie ahead for this area of research.
